# Activated Protein C in Cutaneous Wound Healing: From Bench to Bedside

**DOI:** 10.3390/ijms20040903

**Published:** 2019-02-19

**Authors:** Ruilong Zhao, Haiyan Lin, Lara Bereza-Malcolm, Elizabeth Clarke, Christopher John Jackson, Meilang Xue

**Affiliations:** 1 Sutton Arthritis Research Laboratory, Kolling Institute of Medical Research, Sydney Medical School, Northern Clinical School, University of Sydney, Sydney, NSW 2065, Australia; rzha9073@uni.sydney.edu.au (R.Z.); haiyan.lin@sydney.edu.au (H.L.); lara.bereza-malcolm@sydney.edu.au (L.B.-M.); chris.jackson@sydney.edu.au (C.J.); 2 Murray Maxwell Biomechanics Laboratory, Kolling Institute of Medical Research, Sydney Medical School, Northern Clinical School, University of Sydney, Sydney, NSW 2065, Australia; elizabeth.clarke@sydney.edu.au

**Keywords:** activated protein C, wound healing, cytoprotection, cell culture, animal models, clinical trials

## Abstract

Independent of its well-known anticoagulation effects, activated protein C (APC) exhibits pleiotropic cytoprotective properties. These include anti-inflammatory actions, anti-apoptosis, and endothelial and epithelial barrier stabilisation. Such beneficial effects have made APC an attractive target of research in a plethora of physiological and pathophysiological processes. Of note, the past decade or so has seen the emergence of its roles in cutaneous wound healing—a complex process involving inflammation, proliferation and remodelling. This review will highlight APC’s functions and mechanisms, and detail its pre-clinical and clinical studies on cutaneous wound healing.

## 1. Background

### 1.1. Production and Activation

**Figure 1:**
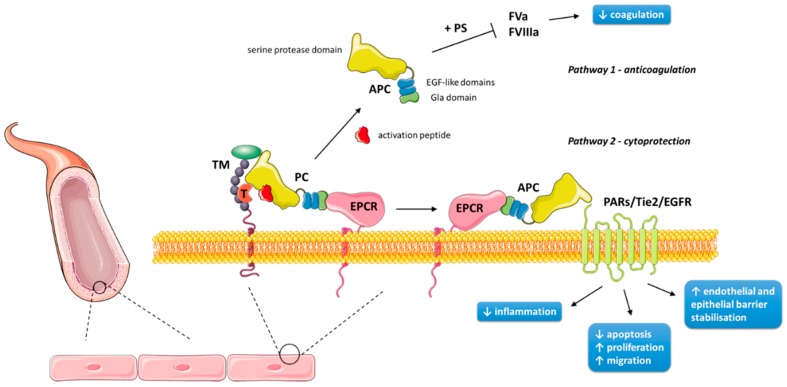
Mechanism of protein C activation and actions on the surface of human endothelial cells. Protein C (PC) is bound to an endothelial protein C receptor (EPCR) on the surface of endothelial cells, where it is activated by thrombin (complexed with thrombomodulin) cleavage of its activation peptide. Activated protein C (APC) is then either released, where it participates in negative feedback of the coagulation cascade (pathway 1), or presented to cleave PARs/Tie2/EGFR in order to exert its cytoprotective effects (pathway 2). Pathway 2 also occurs on the surface of keratinocytes. EGFR: endothelial growth factor receptor; FVa: activated factor V; FVIIIa: activated factor VIII; PAR: protease-activated receptor; PS: protein S; T: thrombin; TM: thrombomodulin. Figure was produced using Servier Medical Art (https://smart.servier.com).

Protein C (PC) was first noted to play a role in anticoagulation in 1960 [1], but was not isolated until 1976, when it was determined to be a vitamin K-dependent zymogen [2]. Its synthesis begins predominantly in liver cells from the human *PROC* gene on chromosome 2 (2q13-14) [3]. There it is translated from nine exons to a single chain precursor molecule, composed of a signal peptide and a propeptide [4]. Key post-translational modifications include β-hydroxylation at Asp71; N-linked glycosylation at residues 97, 248, 313 and 329; and γ-carboxylation of nine glutamic acid residues to form the Gla domain of the amino terminus [3]. Upon secretion, the precursor is cleaved to produce one light chain (21 kDa) and one heavy chain (41 kDa) connected by a disulfide bond, creating the mature heterodimer that is PC [4]. PC consists of several moieties: a Gla domain, an aromatic segment and two epidermal growth factor (EGF)-like domains within the light chain, and a serine protease domain and activation peptide within the heavy chain [5]. It is primarily in this form that PC circulates as a zymogen in the plasma at 70 nM, whereas its activated form is present at 40 pM [6]. Activated protein C (APC) is generated by thrombin cleavage, removing the activation peptide. This process occurs primarily on the endothelial cell surface, and is strongly promoted by endothelial protein C receptor (EPCR) and the formation of a thrombin–thrombomodulin complex [7,8]. Human epidermal keratinocytes have also been found to express PC, EPCR, thrombomodulin, and other related receptors (described below) involved in PC’s actions and activation [9,10,11,12]. Figure 1 provides a schematic representation of this activation on endothelial and epithelial cells.

APC’s pleiotropic activities make it an attractive candidate for potential roles in the treatment of complex disorders, including sepsis [13], ischaemic stroke [14], and chronic wounds [15]. APC has two main classes of functions: anticoagulation and cytoprotection.

### 1.2. Anticoagulation Pathway

APC performs its anticoagulant effects along with cofactors, including factor V, high density lipoprotein, anionic phospholipids, glycosphingolipids, and in particular, protein S—the Gla domain mediating the cofactors’ Ca^2+^-dependent interactions [16,17]. On the surface of negatively charged membrane surfaces, such as platelets, APC irreversibly proteolytically cleaves coagulation factors Va and VIIIa (Figure 1), thus inactivating both intrinsic and extrinsic coagulation cascades [18,19]. An insight into the significance of APC as an anticoagulant is elucidated from the fatal consequences of its homozygous deficiency, which causes neonatal purpura fulminans and its associated skin necrosis; its significance can also be seen from the increased risk of venous thrombosis with APC’s heterozygous deficiency [20].

### 1.3. Cytoprotective Pathways

APC has several cytoprotective properties, which are independent from its anticoagulation pathway [21]. These include anti-inflammatory actions, anti-apoptosis, and endothelial and epithelial barrier stabilisation [3]. Many such properties require EPCR and protease-activated receptor (PAR)-1, a G protein-coupled receptor [22]. EPCR, which itself is anti-inflammatory, binds APC and presents APC to the cleavage site on PAR-1 [23] (Figure 1). Interestingly, PAR-1 is primarily a thrombin receptor; thrombin cleaves PAR-1 much more efficiently than APC, and the thrombin-triggered PAR-1 signalling pathway is pro-inflammatory and endothelial barrier-disruptive [24]. This paradox may be explained by the co-localisation of EPCR and PAR-1 on caveolin-1-enriched membrane rafts or caveolae, boosting the efficiency of PAR-1 activation by APC within these microdomains [25,26], as well as by PAR-1 being shown to be a biased receptor. Thrombin cleaves and activates PAR-1 at the canonical Arg41 site, whereas APC cleaves PAR-1 predominantly at Arg46 in the presence of EPCR [27,28]. The APC-cleaved peptide comprised of PAR-1 residues 47–66 (TR47) has been shown to mimic APC’s cytoprotective properties in vitro and in vivo [27]. 

The anti-inflammatory effects of APC are primarily associated with actions on endothelial cells and leukocytes. APC limits tissue damage through the suppression of the nuclear factor (NF)-κB pathway in endothelial cells. APC also inhibits inflammatory mediators such as tumour necrosis factor (TNF)-α, and down-regulates vascular adhesion molecules, such as intercellular adhesion molecule-1, reducing leukocyte adhesion and infiltration of tissues [29,30]. In addition, APC maintains endothelial barrier function and reduces chemotaxis [21]. Similarly, APC also diminishes leukocyte release of cytokines, such as macrophage inflammatory protein-1α, monocyte chemoattractant protein-1 (MCP-1), and TNF-α, to potentially attenuate systemic inflammatory responses [31,32]. In rheumatoid synovial fibroblasts and monocytes, APC further suppresses NF-κB activation and TNF-α production, while upregulating anti-inflammatory matrix metalloprotein (MMP)-2 and downregulating pro-inflammatory MMP-9 [33]. Moreover, a recent study has further indicated that APC suppresses nucleotide-binding oligomerization domain-like receptor protein 3 (NLRP3) inflammasome activation via PAR-1 in mice macrophages and cardiac and renal tissue [34]. These data identify APC as a viable and critical target of anti-inflammatory therapy.

APC up-regulates around 20 genes engaged in anti-inflammatory and anti-apoptotic pathways, and down-regulates around 20 genes in pro-inflammatory and pro-apoptotic pathways [21]. While many of these mechanisms are not well understood, it is believed that they at least partly involve the inhibition of transcription factor activity. Of note, Joyce et al. [35] have shown that APC suppresses expression of *p50* and *p52* NF-κB subunits, which is implicated in cytokine signalling and TNF-α-dependent inflammatory pathways. Furthermore, APC augments anti-apoptotic gene products related to Bcl-2, and suppresses pro-apoptotic *p53* and *Bax* expression [36,37]. 

APC has demonstrated inhibition of apoptosis in a number of cells. Its activity is dependent partly on modulation of gene expression, and partly on the direct inhibition of apoptotic mediators, such as caspases-3 and -8 [21,35]. When brain endothelial cells are under hypoxic stress, APC reduces pro-apoptotic p53 and Bax, but maintains levels of protective Bcl-2, thereby minimising stimulation of the intrinsic apoptotic pathway [37]. In human skin keratinocytes, APC suppresses apoptosis by preventing the activation of caspase-3 [38]. On the flipside, APC stimulates angiogenesis, and both the proliferation and migration of human keratinocytes [38,39].

Endothelial cells serve as a barrier between the intravascular compartment and the interstitium, and breakdown of this barrier is a key pathogenic factor in inflammation. APC’s barrier protective mechanisms act through EPCR-dependent PAR-1 activation in two main ways. Firstly, APC stimulates sphingosine kinase-1 to form sphingosine-1-phosphate (S1P) from sphingosine [40]. When activated by S1P, sphingosine-1-phosphate receptor 1 (S1P1) mediates second messengers Rac (protective) and Rho (destabilising) to stabilise the cellular cytoskeleton, reducing endothelial permeability [41]. Secondly, APC employs the angiopoietin/Tie2 axis to up-regulate zona occludens-1 and smooth muscle cell migration, significantly reducing human umbilical vein endothelial cell (HUVEC) permeability [42]. Additionally, PC makes direct contributions to vascular membrane formation via the stimulation of type IV collagen and MMP-2 [43]. Finally, through similar pathways, APC has also been shown to promote barrier functions of the skin, as well as the intestinal and alveolar epidermis [12,44,45].

In addition to EPCR and PAR-1, studies have revealed other key receptors that play a major role in APC-mediated signalling of different cells, such as S1P1, several integrins, PAR-2, PAR-3, apolipoprotein E receptor 2 (ApoER2), glycoprotein Ib, CD11b, Tie2, and EGF receptor (EGFR) [30,46]. For example, Xue et al. [12] show that when activated on confluent keratinocytes, PAR-1’s coupled G protein transactivates EGFR, which further activates the Tie2 receptor; this action enhances PI3K/Akt and inhibits ERK to stimulate junctional complexes and reduce keratinocyte permeability. Similarly, APC stabilises the endothelial barrier by activating PAR-1 and Tie2 while bound to EPCR [42], or by binding directly to Tie2 [47]. APC can also act independently of EPCR by binding to integrin CD11b/CD18 on macrophages to facilitate anti-inflammatory actions [48], or by inhibiting apoptosis in podocytes through the proteolytic activation of PAR-3 [49]. APC-ameliorated nephropathy is achieved by PAR-1 and PAR-3 in podocytes [50]. In human lymphocytes, APC stimulates phosphorylation of EGFR to arrest the lymphocytes’ directed migration [51]. In MDA-MB-231 cancer cells, APC requires three receptors—EPCR, PAR-1, and EGFR—to promote cell invasion [52]. APC suppresses human osteoclast differentiation mainly by inhibiting the formation of multinucleated cells via EPCR, PAR-1, S1P1, and ApoER2 [53]. Finally, the wound promoting effects of APC on mouse full-thickness wounds depends on PAR-2 activity [54], and APC signals via PAR-2 and PAR-3 to expand regulator T-cells, mitigating graft-versus-host disease in mice [55].

## 2. Active Protein C in Wound Healing

### 2.1. Cell Culture

**Figure 2:**
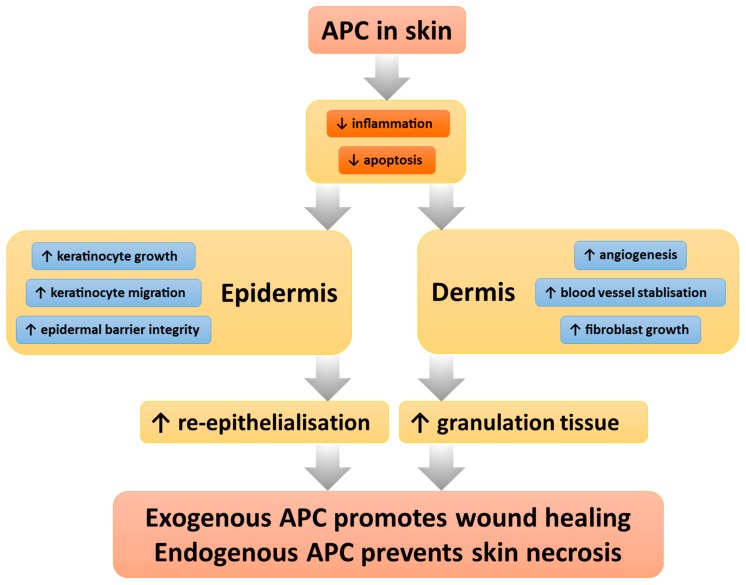
APC exerts its protective actions on major cellular components of the skin to ensure normal homeostasis.

Keratinocytes, endothelial cells, and fibroblasts are the major cell types in the skin, and play critical roles in wound healing. Keratinocytes and endothelial cells form important functional barriers, and all three cell types express a plethora of cytokines and growth factors involved in wound healing. APC acts on these major cellular components to ensure their appropriate functions in cutaneous wound healing (Figure 2).

Keratinocytes represent the major cellular component of the epidermis, and are responsible for maintaining structure and homeostasis of the epidermal barrier [56]. Once thought to be synthesised exclusively by the liver and endothelial cells, more recent evidence shows that keratinocytes also express PC mRNA and protein, and exhibit APC activity [9]. In cultured human keratinocytes, APC promotes proliferation, while gene silencing of PC increases apoptosis three-fold [9]. Importantly for wound healing, APC dose-dependently stimulates keratinocyte migration, possibly by its stimulation and activation of MMP-2 [38], which also has anti-inflammatory properties. MMP-2 degrades collagen present in the basement membrane, which is crucial in the invasive processes of re-epithelialisation and in angiogenesis [57]. Finally, in keratinocyte monolayers, APC decreases permeability to enhance barrier function by up-regulating tight junction proteins and redistributing them to cell–cell contacts, via the signaling pathway described above [12].

Endothelial migration and proliferation are vital in generating new vessels during the proliferative phase of healing. APC up-regulates gene and protein expression of angiogenic factors in several cultured human cells [57]. In particular, APC enhances MMP-2 and MCP-1 in fibroblasts and HUVECs, and vascular endothelial growth factor (VEGF) in keratinocytes and fibroblasts [58,59]. Using a chick embryo chorioallantoic membrane assay, Jackson et al. [58] showed that APC stimulates massive formation of fine capillary vessels, as well as a marked proliferation of the ectodermal epithelium. Similar to keratinocytes, APC induces endothelial cell proliferation, tube-like structure formation, and migration in vitro [39,59]. As mentioned previously, APC also maintains the essential endothelial barrier function.

Smooth muscle cells have been shown to express functionally active EPCR, potentially contributing to the formation of mature blood vessels [60]. In dermal fibroblasts, which help lay down the provisional wound matrix and guide wound contraction and maturation, APC increases MMP-2, VEGF and MCP-1 in human fibroblasts, although the mechanisms are not clearly understood [58].

### 2.2. Animal Models

**Figure 3:**
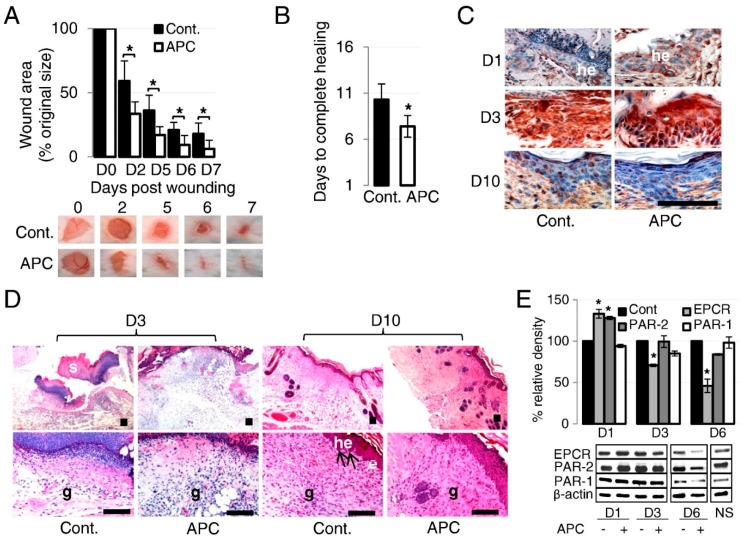
APC accelerates wound healing in wild-type (WT) mice. (**A**) and (**B**) Full-thickness 6-mm diameter wounds were made and treated topically with 20 μL of phosphate-buffered saline or APC (10 μg) once a day for three consecutive days. (**A**) Representative photographs of skin wounds on days after APC treatment. The percentage of wound area/initial area was calculated from tracing the wounds, measured with Visitrak. Values are mean ± SD; *n* = 10 wounds. * *p* < 0.05 by paired *t*-test. (**B**) Time to complete wound closure. Values are mean ± SD; *n* = 10 wounds. * *p* < 0.05 by *t*-test. (**C**) Expression of PAR-2 by immunohistochemistry on wounded skin in WT mice (he: hyperproliferative epithelium). Scale bar = 100 μm. (**D**) Hematoxylin and eosin -stained paraffin sections from day 3 and day 10 wounds from WT mice. Arrows indicate the leading edge of the migrating epithelial tongue (e: epithelium; g: granulation tissue; he: hyperproliferative epithelium; s: scab). Scale bar = 100 μm. (**E**) Expression of EPCR, PAR-1, and PAR-2 detected by immunoblotting from homogenate supernatants of wounded skin (NS: normal skin). The band intensity of the protein was normalized with β-actin, and each control was defined as 100%. Values are mean ± SD; *n* = 3. * *p* < 0.05 versus each control on each day by paired *t*-test. Copyright © 2011 American Society for Investigative Pathology. Published by Elsevier Inc. All rights reserved. Reproduced with permission. License number 4526191003342.

The findings on APC’s anti-inflammatory and cytoprotective properties prompted researchers to investigate its action on cutaneous wound healing in experimental animals (Table 1). Following their in vitro work, Uchiba et al. [39] demonstrated that APC induces a corneal angiogenic response in mice comparable to that of VEGF, the most potent angiogenic mediator currently known. Endothelial nitric oxide synthase (eNOS) was essential in APC’s angiogenic pathway, as angiogenesis was not induced in eNOS knockout mice. Jackson et al. [58] showed that a single topical application of APC enhanced healing in full-thickness excisional wounds when compared to the control in both normal and diabetic rats, with no adverse side effects like toxicity or bleeding observed. APC-treated wounds had more blood vessels on day 7, and lower neutrophil infiltration on days 4 and 7; however, the addition of a broad spectrum MMP inhibitor, GM6001, abolished APC’s actions. Their data suggest that APC’s multifaceted promotion of cutaneous wound healing involves at least the stimulation of angiogenesis and inhibition of inflammation. Julovi et al. [54] confirmed these results in C57BL/6J mice (Figure 3), and further used PAR-1 and PAR-2 knockout mice to reveal that PAR-2, but not PAR-1, was necessary for APC to accelerate wound healing; in addition, they confirmed that inhibition of phosphorylated p38 via APC’s cleavage of PAR-2 boosted healing in wild-type mice. Three studies further examined the cutaneous effects of systemic APC—one on flap necrosis and two on burn injuries. In dorsal cutaneous flaps on rats, systemic APC significantly improved flap survival compared to controls, corresponding to increased blood vessel density and muscle cell viability, with fewer inflammatory cells; PCR revealed the modulation of several genes associated with these outcomes [61]. The two burn models appeared to show conflicting results. Nisanci et al. [62] utilised a “comb burn” model with eight 1 cm × 2 cm areas per rat injured by a heated brass block held for 20 seconds, showing positive results for APC treatment. However, Meyerholz et al. [63] injured ten 2 cm × 2 cm areas per rat that ranged from 1 to 14 seconds of heated aluminium branding, revealing deleterious APC treatment effects. The former clearly created smaller but deeper burns with no fluid resuscitation following injury, while the latter had larger and more superficial burns with fluid resuscitation via femoral vein cannulation. It may be that the larger, more superficial burns (most of which were branded with contact times of less than 10 s were not severe enough and were too well resuscitated to show the benefit—a landmark trial had shown that APC’s benefit in severe sepsis was limited to those with Acute Physiologic Assessment and Chronic Health Evaluation (APACHE) II scores of 25 or higher [64]. Furthermore, Nisanci et al. measured perfusion on day 3, compared to Meyerholz et al. who measured just 5 h after injury. However, in the “comb burn” model, APC only increased blood flow in the zone of stasis, and not the actual burned area (taken to be the zone of coagulation), which is potentially congruent with the miniscule perfusion differences in just the more severely burned areas of the latter study. Perhaps systemic APC treatment has a paradoxically negative effect on burn injuries in the acute phase, especially in less severe injuries; however, this effect reverses to become advantageous in the subacute phase. A possible culprit for this difference early on may be found in a syndrome called acute traumatic coagulopathy, where endothelial PC activation plays a central role—exogenous APC exacerbates any rapid anticoagulation and fibrinolysis following severe trauma, which can worsen organ-specific and systemic complications [65]. In large animals, APC has had no effect on macroscopic healing metrics for equine distal limb wounds, but has demonstrated enhanced epithelialisation and angiogenesis histologically [61]. This disparity with small animal studies may be explained by their indirect method of APC application (gauze pad soaked in APC solution); while this method was more practical in horses, optimal local APC may not have been achieved in their wounds. However, recent data from our group in a porcine wound model did show increased healing through a combined topical and subcutaneous APC treatment regime, with no bleeding side effects. Together, these results indicate that APC’s impacts are evident in not only cell culture but also animal wounds, where it is both effective and safe to use.

While in vitro and in vivo investigations can provide valuable insight into specific pathways and a focused approach to the pathophysiology underlying human chronic wounds [66], the lack of concordance with human skin has been cited as a major impediment into translational research. Mice and rats are loose-skinned, and their open wounds heal mostly by contraction—a stark contrast to the lower limbs of humans, a common site for chronic wounds [67,68,69]. However, the unpublished porcine study shows promising results in an animal with 78% concordance rate to human wound healing [70]. Unfortunately, another difficulty presents itself in reproducing comorbid and causative conditions, such as neuropathy, chronic debility or vascular insufficiency in animals, and there is a paucity of truly aged animals to provide precise models of chronic wounds [71]. Nonetheless, the positive results described above have encouraged researchers to pursue small clinical trials in human chronic wounds.

### 2.3. Clinical Trials

**Figure 4:**
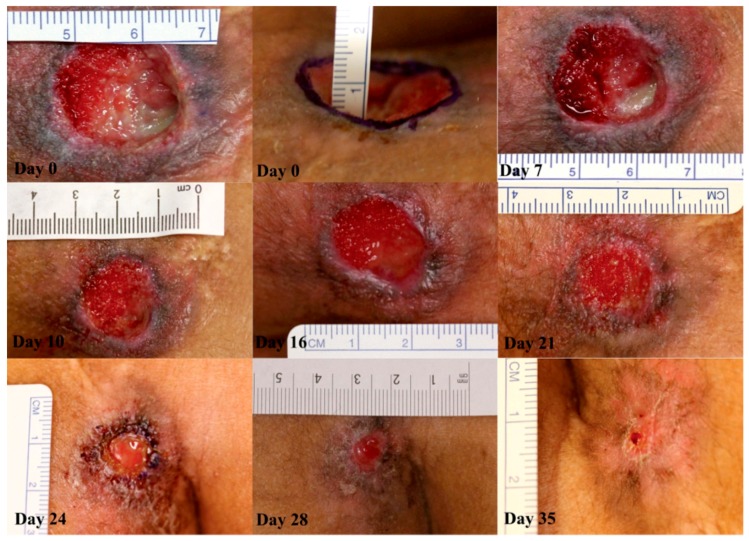
Case 1: sacral pressure ulcer progression through healing after activated protein C (APC) administration. On day 0, the wound had a central depth of 10 mm. At day 7, the wound was 20 mm in diameter. Rapid growth of granulation tissue is apparent from day 7 to day 16. At day 16, the diameter was 18 mm. On day 21, the diameter was 14 mm, and by day 24 the wound was 6 mm in diameter. Therapy was stopped at this point. At day 28, the diameter was 5 mm. Final follow-up at day 35 showed that the wound had healed completely. No images of the follow-up. © 2014 The Authors. International Wound Journal © 2014 Medicalhelplines.com Inc and John Wiley & Sons Ltd. Reproduced with permission. License number 4525880595450.

**Figure 5:**
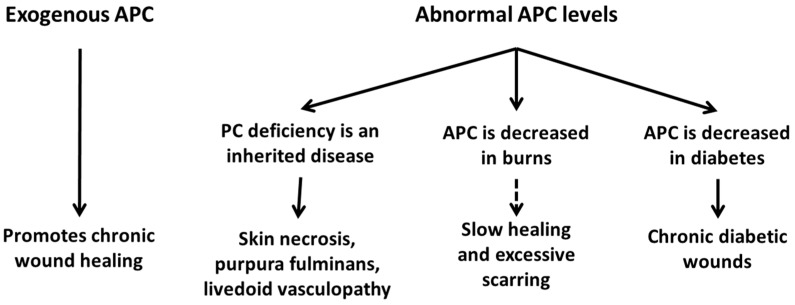
In humans, low APC levels are associated with chronic diabetic wounds, severe burn injuries, and skin necrosis secondary to purpura fulminans. Exogenous APC has been shown to promote the healing of chronic wounds of varying aetiologies.

In addition to the animal experiments, APC’s associations with human non-healing wounds have been reported in six clinical studies (Table 2). Similar to the majority of the animal studies, APC was applied topically in study participants to minimise potential systemic side effects, and the macroscopic endpoints used also included time to complete healing and wound area reduction. With the exception of one study where biopsies were taken, no other clinical studies assessed wound healing metrics microscopically. Firstly, Whitmont et al. [72] conducted an open-label pilot study on four patients with non-healing leg ulcers of various causes for four months or more despite standard wound care, who received weekly topical applications of APC for four weeks. The treatment was well-tolerated, with no significant side effects or complications, and all four patients showed a rapid positive response that was maintained during a four-month follow-up period; more than 80% reduction in wound size was observed overall after eight weeks. The same group later showed that diabetic patients with lower leg ulcers have significantly reduced levels of circulating PC compared to diabetic patients without ulcers, when corrected for age and matched for gender and type of diabetes [73]. The authors proposed that lower plasma PC levels may predispose ulceration in diabetic patients. Wijewardena et al. [74] piloted another study, where four patients with recalcitrant orthopaedic wounds were treated with APC in conjunction with topical negative pressure (TNP). Within one week, all cases showed a clear reduction in wound size and depth, with remarkable increases in granulation tissue; the treated wounds either closed completely or had sufficient granulation tissue to allow for split-thickness skin grafting. APC treatment was well-tolerated and no osteomyelitis was seen in the long-term follow-up. These promising early results necessitated a randomised, placebo-controlled, double-blind trial to rigorously determine the efficacy of APC. Whitmont et al. [75] produced such a study involving 12 diabetic patients with lower leg ulcers, randomly assigned to receive topical APC or physiological saline. They reported that APC significantly reduced the wound areas at 20 weeks, with three APC-treated wounds completely healing compared to one saline-treated wound. Wound edge skin biopsies showed that APC treatment decreased inflammatory cell infiltration and increased vascular proliferation, similar to the histological results of the small animal studies. They also reported reduced patient stress scores following APC treatment, as assessed by the Cardiff Wound Impact Questionnaire, demonstrating improved quality of life. Two further case series have been published, each involving two patients, determining the efficacy of APC for ulcers caused by pyoderma gangrenosum in one study, and severe chronic pressure sores in the other [76,77]. APC was injected subcutaneously and applied topically combined with TNP, respectively; both methods led to clinical improvements and reductions in wound size (Figure 4). Although these studies conducted so far have been small, such promising results in refractory wounds, even those of dissimilar aetiologies, paint an encouraging picture of APC as a safe wound-healing agent, and provide supporting evidence for future larger clinical trials. Recently, a larger observational study in burn patients has been completed by our group, where it was found that plasma PC levels rose over time with patient recovery, and that low day 0 plasma PC levels predicted for worse clinical outcomes. The known effects of APC in human skin are summarised in Figure 5.

Although the various human chronic ulcers differed in pathophysiology to the animal acute wounds, both clinical and preclinical studies found that APC exerted effects in the epidermis, where it promoted re-epithelialisation, as well as in the dermis, where it encouraged granulation tissue formation while reducing inflammatory infiltrate. The consistent positive results add weight to the rationale for APC’s role in wound healing, originally formed through in vitro experiments (Figure 2).

By suppressing inflammation, inducing angiogenesis, and re-epithelialisation, APC is also likely to minimise scar formation, in addition to hastening wound healing. The obvious benefit is for burn victims and those susceptible to keloid scarring [30]. However, the application of APC may also hold great potential for any surgical wound, where enhancements in healing times and wound aesthetics would constitute improved clinical outcomes and patient satisfaction.

## 3. Active Protein C in Other Diseases

APC may also have therapeutic benefits in a number of other diseases, including sepsis [13], central nervous system injury [78], ischaemic stroke [14], Alzheimer’s disease [79], acute kidney injury [80], lung disorders [81], acute pancreatitis [82], type I diabetes [83], rheumatoid arthritis [33,84], and cancer [85] (Table 3). Besides its obvious use in homozygous PC deficiency, APC has only been approved for severe sepsis. PC levels are known to be a strong prognostic factor in septic patients [86]. Due to this, and its pleiotropic anticoagulative and cytoprotective properties, APC has been long been suggested to treat sepsis. Activated drotrecogin alpha, or recombinant human APC (rhAPC), was the first U.S. Food and Drug Administration (FDA)-approved drug for the treatment of severe sepsis in 2001. Evidence from two large clinical trials suggested rhAPC reduces mortality, but may have increased the risk of bleeding [64,87]. However, since then, APC has been the subject of much controversy with regards to its efficacy and safety in certain patient populations, such as those with single organ dysfunction who have recently undergone surgery (within 30 days) [88,89]. Published in 2012, the results of another large trial showed that rhAPC did not significantly reduce mortality [90]. This resulted in Eli Lilly, a global pharmaceutical company that produced rhAPC, to voluntarily withdraw their drug from public use worldwide in 2011. A series of Cochrane reviews in 2011 and 2012 concluded that despite the scientific rationale, there is insufficient data for APC’s use in septic neonates, children or adults, and advised against its further promotion [91,92,93]. However, further developments in mutant APC, without its anticoagulant properties, have reinvigorated research into its potential benefits.

It should be emphasised that no side effects, including toxicity or bleeding, were noted in the animal and human wound studies. This is likely resultant from local applications of APC as opposed to systematic, as in the sepsis studies.

## 4. Engineered Protein C/Active Protein C

APC’s potent anticoagulation activity involves stereospecific interactions with factors Va and VIIIa at both its enzymatic active site and secondary binding sides, called exosites [94]. These exosites differ from the ones needed for binding to EPCR and PAR-1, and thus can be mutated to diminish the anticoagulation effects of APC while preserving its cytoprotective properties [95,96]. Mosnier et al. [95] generated two variants—229/230-APC (RR229/230AA) and 3K3A-APC (KKK191-193AAA—in which a cluster of positive residues were replaced by two and three alanine residues, respectively, restructuring a crucial positive region for binding factor Va. These two mutations severely reduced APC’s anticoagulation activity but retained normal anti-apoptotic actions. The same group later showed that combining the two mutations formed a new variant—5A-APC—with even less anticoagulation activity (<0.1% FVa inactivation compared to wild type APC), but normal cytoprotective activity in cells [97]. Bae et al. [98] confirmed the distinction between the anticoagulant and cytoprotective exosites of APC by engineering a disulphide bond between two β-sheets, which stabilised the functionally critical Ca^2+^-binding 70–80 loop, yielding similar results to the previous two studies. Various other mutations have been characterised to elucidate the underlying mechanisms to APC’s pleiotropic functions; the numerous mutational studies of APC are summarised by Sarangi et al. [99]. These have included changes in anticoagulation, cytoprotection, cofactor dependency, and half-life, among others. Specifically, the mutations that minimise anticoagulation produced variants that have equivalent or sometimes even greater beneficial effects than wild-type recombinant APC, as demonstrated in animal models of stroke [100,101,102,103,104,105], traumatic brain injury [106], amyotropic lateral sclerosis [107], endotoxaemia and sepsis mortality [108,109,110], myocardial and liver ischaemic/reperfusion injury [111,112,113], and *Pseudomonas aeruginosa* pneumonia [114]. A notable example (APC-L38D/N329Q) was generated with five-fold enhanced endothelial barrier protective function, as well as 30-fold improved anti-apoptotic function [115]. Important examples pertaining to cytoprotection are summarised in Table 4. A synthetic 20-mer peptide (TR47) consisting of the APC-cleaved N-terminus of PAR-1, has further been described with pharmacological actions resembling those of APC [27]. 

Mosnier et al. [110] generated another APC mutant (E149A-APC) with greatly reduced cytoprotective effects but superior anticoagulant activity, which has been useful for proof-of-concept studies and for antithrombotic indications. Experiments comparing E149A-APC to wild-type APC and 5A-APC further emphasise that APC’s anticoagulant property is unnecessary to confer its cytoprotective activities [105,110].

Notably, 3K3A-APC has successfully completed both Phase I and II trials for ischaemic stroke [116,117], but is yet to be assessed in wound healing. Recent work from our group has shown 3K3A-APC accelerates excisional wound healing in mice and pigs, including promoting collagen maturation in pigs. The FDA is currently being approached for approval to conduct a Phase II trial on diabetic foot ulcers.

Although these variants are yet to be assessed in the field of clinical wound healing, the engineered APCs are expected to exert similar anti-inflammatory and cytoprotective actions in the skin as they have demonstrated in other tissue and disease models. Furthermore, the potential side-effect of APC to increase bleeding in wounds has not been seen in studies so far, and appears to be circumvented by both engineered variants and topical and subcutaneous applications, instead of systemic.

## 5. Conclusions

APC’s multimodal actions make it an extremely useful and versatile agent in a wide range of disease areas. Since the start of the millennium, its role in wound healing has embarked on a journey of discovery from cell culture to animal studies, and to small clinical trials. APC’s remarkable wound healing properties have been extensively demonstrated, and its mechanisms thoroughly elucidated. Its future now may well lie in one of its engineered variants, with greater cytoprotection and fewer anticoagulation properties. Of these, 3K3A-APC is poised to take the next step in human clinical trials.

## Figures and Tables

**Table 1:** APC in animal wound models.

Study	Animal	Wound Model	Dosage	Mode of Administration	Duration of Administration	Effects
Uchiba et al. [39]	Male eight-week-old C57BL/6 mice and eNOS KO mice	Corneal angiogenesis	Not reported	Slow-release pellets implanted in corneal stroma	6 days	↑ corneal angiogenic response
Jackson et al. [58]	Normal and diabetic Sprague–Dawley rats	Full-thickness excisional wounds	5 µg, 20 µg and 40 µg/wound	Topical	Single dose	↑ healing in both normal and diabetic rats ↓ inflammation ↑ angiogenesis
Bezuhly et al. [36]	Male Sprague–Dawley rats	Cranially based dorsal cutaneous flap	25 μg/kg	Tail vein injection	3 injections (early pre-, late pre-, or post-operatively; 3 h post-operatively; and 24 h post-operatively)	↑ ischaemic flap survival ↓ inflammation ↑ angiogenesis ↑ muscle cell viability ↓ mRNA of ICAM-1 and TNF-α ↑ mRNA of EGR-1, VEGF, and Bcl-2
Nisanci et al. [62]	Male Sprague–Dawley rats	Burn injury	100 μg/kg	Tail vein injection	One injection two hours after burn injury	↑ tissue perfusion ↓ area of skin necrosis in zone of stasis
Meyerholz et al. [63]	Male Sprague–Dawley rats	Burn injury	24 μg/kg/h	Microinfusion pump through proximal femoral vein	5 h from burn creation to animal sacrifice	↓ tissue perfusion ↑ burn depth ↑ inflammation ↓ apoptosis
Julovi et al. [54]	Male C57BL/6J WT, and PAR-1 and PAR-2 KO mice	Full-thickness excisional wounds	10 µg/wound	Topical	Once per day for 3 days	↑ healing in WT and PAR-1 KO mice ↓ inflammation ↑ angiogenesis ↑ re-epithelialisation. Negligible effect in PAR-2 KO mice
Bischofberger et al. [61]	Standardbred geldings	Full-thickness excisional wounds	190 µg/wound	APC solution-soaked gauze pad	4 h applications on days 1, 3, 6 and 9	No effect on wound size, rate of healing, or overall time to heal ↑ angiogenesis ↑ re-epithelialisation

APC: activated protein C; Bcl: B-cell lymphoma; EGR: early growth response protein; eNOS: endothelial nitric oxide synthase; ICAM: intercellular adhesion molecule; KO: knockout; PAR: protease-activated receptor; mRNA: messenger ribonucleic acid; VEGF: vascular endothelial growth factor; WT: wild-type; ↑ = increased; ↑ = decreased.

**Table 2:** APC in Clinical Trials.

Study	Study Type	Wound Aetiology	Dosage	Mode of Administration	Duration of Administration	Effects
Whitmont et al. [72]	Open-label pilot study	Venous (*n* = 1), arteriovenous (*n* = 1), diabetic/neuropathic (*n* = 1), diabetic (*n* = 1)	200 µg/mL solution	Topical: APC solution was injected into wound space until level with skin surface (max. 1.6 mL was sufficient)	Once per week for four weeks	All four patients showed steady progress in healing over an eight-week treatment/follow-up period, with a pooled reduction of 80% in wound size
Wijewardena et al. [74]	Open-label pilot study	Recalcitrant orthopaedic wounds (*n* = 4)	400 µg/mL solution at roughly 1 mL/cm^2^	Topical and subcutaneously (max. 3 mL)	Twice per week until complete wound closure or sufficient improvement for surgical intervention	Two wounds healed completely—one allowed for split-thickness skin graft, one allowed for primary closure
Whitmont et al. [73]	Case-control	Neuropathic (*n* = 14), ischaemic (*n* = 10), mixed neuropathic/ischaemic (*n* = 11), venous (*n* = 1), all in diabetic patients	N/A	N/A	N/A	Ulceration in diabetic patients is correlated to lower plasma PC levels
Kapila et al. [76]	Open-label pilot study.	Pyoderma gangrenosum (*n* = 2).	400 µg in 1 mL solution.	Subcutaneously.	Once per week for six weeks.	APC reduced wound area by 78.9% and 70.0% in the two patients, respectively, and pain scores by 100% in both.
Wijewardena et al. [77]	Open-label pilot study.	Pressure ulcers (*n* = 2).	400 µg/mL and 200 µg/mL solutions, respectively.	Topical: APC solution was injected into wound space until level with skin surface (max. 8 mL of the 200 µg/mL solution).	Twice per week for 24 and 64 days, respectively.	Both wounds healed completely by day 35 and 80 respectively.
Whitmont et al. [75]	Randomised controlled trial.	Control: neuropathic (*n* = 4), venous (*n* = 2). APC: neuropathic (*n* = 3), venous (*n* = 3).	400 µg/mL solution.	Topical, APC solution or saline control was injected into wound space until level with skin surface.	Twice per week for 6 weeks.	APC reduced wound area to 36.8 ± 16.4% of week 0 levels at 20 weeks, while control had no significant difference.

**Table 3:** Examples of APC’s therapeutic benefits in other diseases.

Study	Disease
Yamauchi et al. [78]	Spinal cord ischaemia
Cornet et al. [81]	Acute lung injury and acute respiratory distress syndrome
Gupta et al. [80]	Acute kidney injury
Shankar-Hari and Wyncoll [82]	Acute pancreatitis
Spek and Arruda [85]	Cancer
Xue et al. [83]	Type I diabetes
Li et al. [79]	Alzheimer’s disease
Zhang [13]	Sepsis
Amar et al. [14]	Ischaemic stroke
Xue et al. [84]	Rheumatoid arthritis

**Table 4:** Notable examples of mutational studies for cytoprotection.

Study	Name	Mutation	Effects
Mosnier et al. [95]	229/230-APC	Alanine mutations of FVa-binding residues (RR229/230AA)	Reduced anticoagulant activity, with normal anti-apoptotic functions
Mosnier et al. [95]	3K3A-APC	Alanine mutations of FVa-binding residues (KKK191_ 193AAA)	
Mosnier et al. [97]	5A-APC	RR229/230AA and KKK191_ 193AAA	
Bae et al. [98]	No name given	Disulfide bond between two β-sheets (Cys^67^-Cys^82^)	
Ni Ainle et al. [115]	APC-L38D/N329Q	Elimination of an *N*-linked glycan attachment site (L38D/N329Q)	Reduced anticoagulant activity, and improved endothelial barrier protective and anti-apoptotic functions

## Abbreviations

APC	activated protein C
ApoER2	apolipoprotein E receptor 2
EGF	epidermal growth factor
EGFR	EGF receptor
eNOS	endothelial nitric oxide synthase
EPCR	endothelial protein C receptor
HUVEC	human umbilical vein endothelial cell
MCP	monocyte chemoattractant protein
MMP	matrix metalloprotein
NF	nuclear factor
NLRP3	(NOD)-like receptor protein 3
PAR	protease-activated receptor
PC	protein C
rhAPC	recombinant human APC
S1P	sphingosine-1-phosphate
S1P1	sphingosine-1-phosphate receptor 1
TNF	tumour necrosis factor
TPN	topical negative pressure
VEGF	vascular endothelial growth factor
